# Rapid Transit for Physicians

**Published:** 1890-03

**Authors:** 


### RAPID TRANSIT FOR PHYSICIANS.

THE feeling against the use of the bicycle by physicians in their practice is rapidly passing away, and we are confident that it will cease entirely as soon as its advantages are clearly appreciated. In speaking of the bicycle in this connection,we, of course, refer to the pattern known as the rear driver, or "Safety." The high wheel, for obvious reasons, is not practical. Aside from the questions of health and pleasure—no small items of consideration to the busy physician—the one great advantage of the Safety bicycle is that it gives a means of rapid transit ; it economizes time. In a city like Buffalo, with its many miles of smooth pavements, this applies especially, but even over the rough pavements it can be ridden rapidly after a little experience. Other points in favor of the wheel are, that it is easily mastered ; it is inexpensive, after the first cost its maintenance being very little ; it is always ready, an important point, particularly at night. Did patients appreciate how much more rapidly the doctor could come to their aid in cases of sudden illness, they would have no further prejudice against the bicycle. It can be used at all seasons of the year with some little thought to the subject of dress, and in all kinds of weather, excepting, perhaps, on very rainy days. One more point may be mentioned. The bicycle is less tiresome than any form of transit with the horse for the motive power. From actual experience, we can say that we were less weary and more able to attend to what was at hand after riding fifty miles on a bicycle, than after going the same distance in a buggy.

We desire to call attention to one of our advertising pages referring to the Columbia Bicycles. The "Safety" of this name is well adapted to a doctor's needs. They are made by the well-known Pope Manufacturing Company, the pioneer in the construction of wheels in this country. Their workmanship is of the highest order; their guarantee unquestioned. Those contemplating purchasing a bicycle would do well to examine the Columbias before buying. Their agent in this city is Mr. J. H. Isham, No. 301 Main street.

